# Granulomatosis With Polyangiitis in an Autistic Patient With Depression: Challenges in Diagnosis and Management

**DOI:** 10.7759/cureus.73037

**Published:** 2024-11-05

**Authors:** Aashir Javed, Sviatlana Zhyzhneuskaya, Narmeen Khalid, Hanaa Rajabally

**Affiliations:** 1 General Internal Medicine, University Hospital of North Durham, Durham, GBR; 2 Diabetes and Endocrinology, University Hospital of North Durham, Durham, GBR; 3 Gastroenterology, University Hospital of North Durham, Durham, GBR; 4 Rheumatology, University Hospital of North Durham, Durham, GBR

**Keywords:** antineutrophil cytoplasmic antibody (anca) associated vasculitis (aav), autism spectrum disorder (asd), granulomatosis with polyangiitis (gpa), rituximab, small vessel vasculitis

## Abstract

A male patient in his 30s with autism and depression presented to the emergency department with joint pain, a petechial/purpuric rash, sputum production, hemoptysis, and epistaxis. His mother reported a family history of autoimmune conditions. Examination revealed a non-blanching petechial/purpuric rash and tenderness in the feet and ankles. Elevated inflammatory markers, non-blanching rash, previous history of meningitis, and a chest X-ray with patchy opacifications with new oxygen requirements prompted empirical antibiotics and steroids to cover for a chest and possible central nervous system (CNS) infection. Despite antibiotics, inflammatory markers kept rising, necessitating a switch to broad-spectrum antibiotics with subsequent clinical improvement. Later, a positive anti-proteinase 3 antibody test and a CT chest scan showing bilateral ground glass opacities confirmed granulomatosis with polyangiitis (GPA). Management included a weaning course of steroids, proton pump inhibitors, and rituximab infusions. This case highlights the need to consider autoimmune conditions like GPA in autistic patients with multi-system involvement.

## Introduction

Anti-neutrophil cytoplasmic antibody (ANCA)-associated vasculitides (AAVs) are diseases characterized by inflammation of blood vessels, endothelial injury, and tissue damage. There are three types of small-vessel vasculitis, namely granulomatosis with polyangiitis (GPA), microscopic polyangiitis (MPA), and eosinophilic granulomatosis with polyangiitis (EGPA), previously known as Churg-Strauss syndrome) [1].

Granulomatosis with polyangiitis is predominantly associated with PR3-ANCA, and its clinical features typically include sino-nasal disease, lower respiratory tract involvement with pulmonary hemorrhage, granulomatous inflammation, and glomerulonephritis [2]. Between 20% and 50% of patients can demonstrate neurological involvement, including but not limited to, mononeuropathies, cranial neuropathies, cerebrovascular events, meningitis, and seizures [3].

The management of GPA can be particularly challenging when it coexists with neurodevelopmental disorders such as autism spectrum disorder (ASD) and psychiatric conditions like depression. Autism spectrum disorder is a highly heterogeneous neurodevelopmental disorder characterized by behavioral, social, and cognitive deficits [4]. Autism spectrum disorder can complicate the clinical picture and treatment adherence, necessitating a tailored, multidisciplinary approach.

This case report discusses the complex diagnostic and therapeutic challenges exhibited by a young male with ASD and depression who presented with a variety of symptoms on the background of a pre-existing GPA, which was not diagnosed during previous two hospital admissions that were managed as meningitis. It highlights the importance of considering GPA in patients with atypical systemic symptoms, with vasculitis being a mandatory differential diagnosis in a patient presenting with meningitis symptoms and non-blanching petechial/purpuric rash. It also highlights the need for a comprehensive and patient-centered management strategy.

## Case presentation

**Figure 1:**
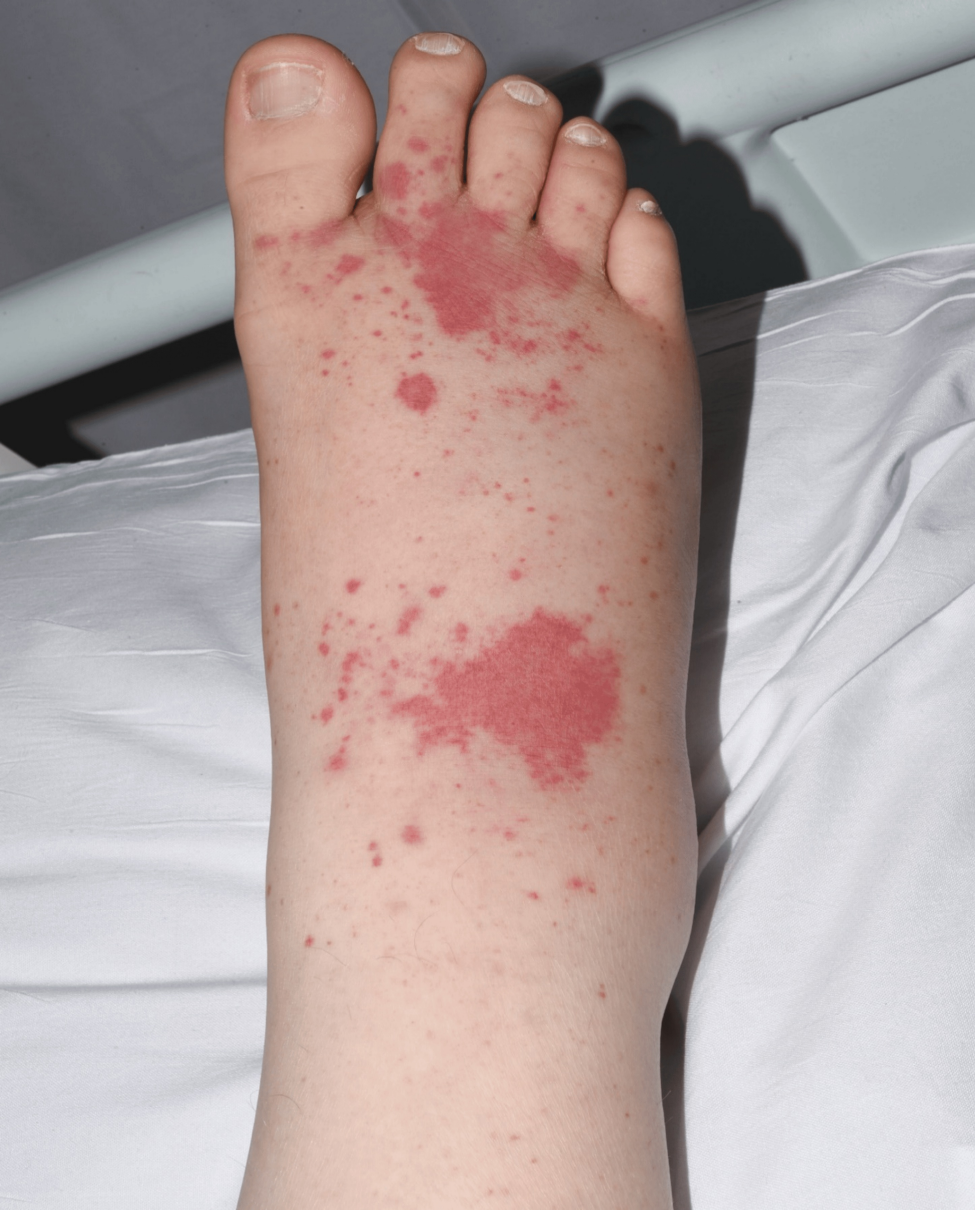
A petechial purpuric rash characterized by small, non-blanching red spots of different sizes on the skin is noted. These spots are due to minor bleeding from broken capillary blood vessels and range from pinpoint size to several millimeters in diameter.

A male patient in his 30s with a medical history of autism and depression presented to the hospital with an acute onset of pain in multiple joints along with a purpuric/petechial rash involving both feet and ankles (Figure 1). His mother provided most of the history, as the patient found it hard to describe his symptoms. As a result, it was challenging to obtain a full, detailed history in one go. Due to autism, the patient described his symptoms in phases when various clinicians reviewed him on different days, resulting in us finding out about new symptoms as the treatment progressed at the hospital. Initially, the pain started in his shoulder and then involved his knees two days later. He had difficulty walking due to the pain. The patient’s mother stated that he had two episodes of meningitis as a child, and a similar-looking rash was seen in both of these episodes. The patient denied headache and neck stiffness. Upon further questioning, it was noted that the patient had been experiencing on-and-off sputum production along with some hemoptysis and epistaxis. His family history was positive for rheumatoid arthritis and scleroderma.

The patient’s initial vital signs showed a blood pressure of 142/88 mmHg, a heart rate of 105 beats per minute, a temperature of 37.3°C, and an oxygen saturation of 96% on room air. On examination, his cardiopulmonary, neurological, and gastrointestinal systems were unremarkable. Examination of feet and ankles revealed tenderness, swelling, warmth, and a petechial/purpuric rash on the dorsal surface, which was non-blanching. The knees were normal on examination; however, the range of motion was restricted due to pain.

**Figure 2:**
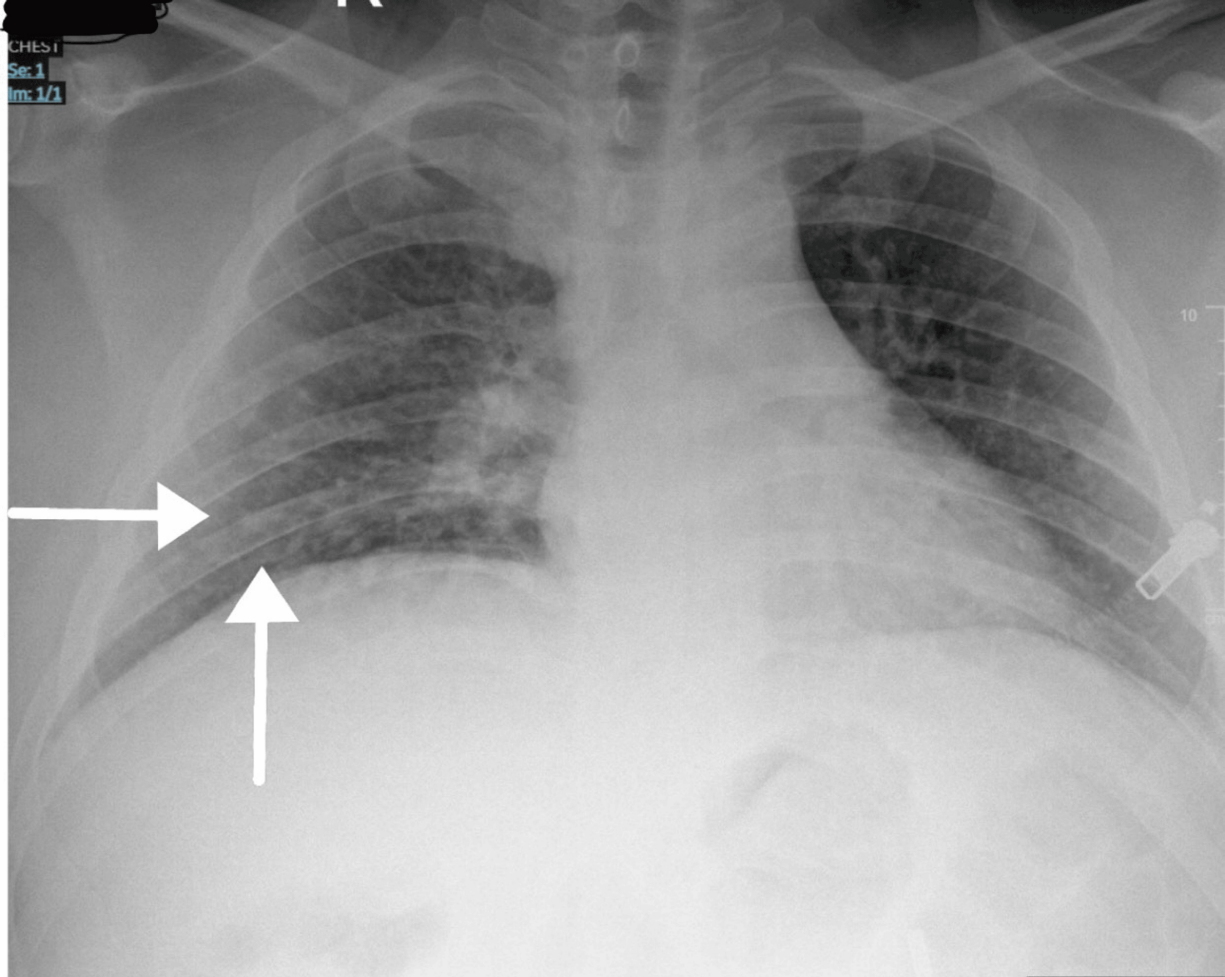
This chest X-ray image reveals areas of patchy opacification in the mid and lower zones of the right lung. These opacifications appear as irregular, ill-defined areas of increased density compared to the surrounding lung tissue.

The following investigations were ordered: a chest radiograph, full blood count, urine and electrolytes, C-reactive protein (CRP), vasculitis screen, and connective tissue antibody screen. Chest radiograph showed patchy peri-bronchoalveolar airspace opacification in the right mid-to-lower zone (Figure 2), while blood tests revealed raised inflammatory markers (white cell count: 14.7 x 10^9/L, neutrophils: 11.3 x 10^9/L, CRP: 98 mg/L). Therefore, the patient was started on empirical intravenous antibiotics to cover the central nervous system (CNS) and chest infection. Additionally, the patient was started on prednisolone 40 mg once daily. A lumbar puncture was planned to rule out meningitis and encephalitis, which the patient subsequently refused. Dermatology advice was sought regarding the rash, who deemed it secondary to the underlying infection.

**Figure 3:**
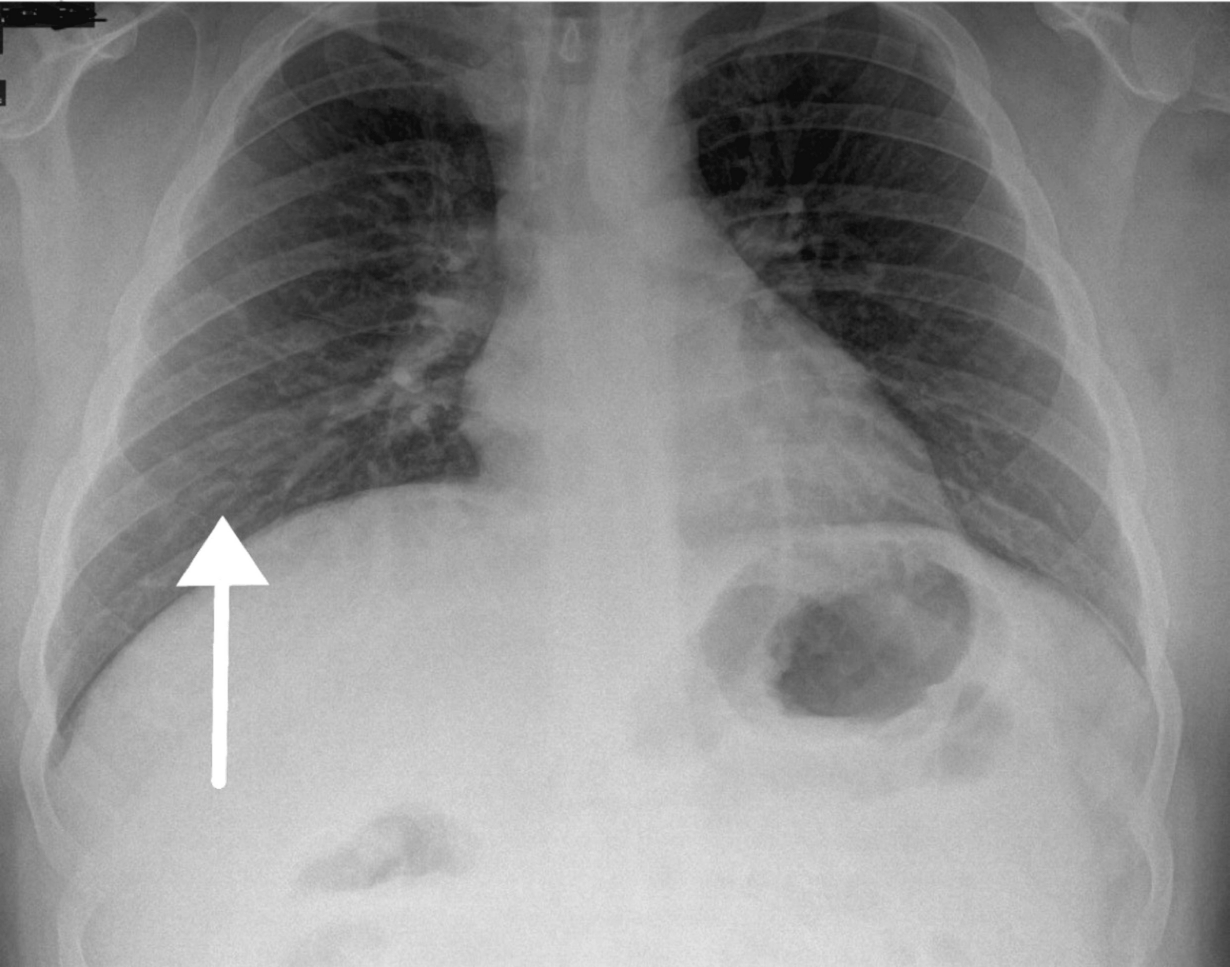
The second chest X-ray showed improvement compared to the previous X-ray.

The patient’s inflammatory markers showed a gradual rise over the next two days as his white cell count increased to 14.2 x 10^9/L while his CRP increased to 167 mg/L. Therefore, the patient was switched to an intravenous broad-spectrum antibiotic. Following this switch, the inflammatory markers showed significant improvement, and the rash on his feet and ankles also resolved. Subsequent chest radiographs (Figure 3) also showed significant improvement.

**Figure 4:**
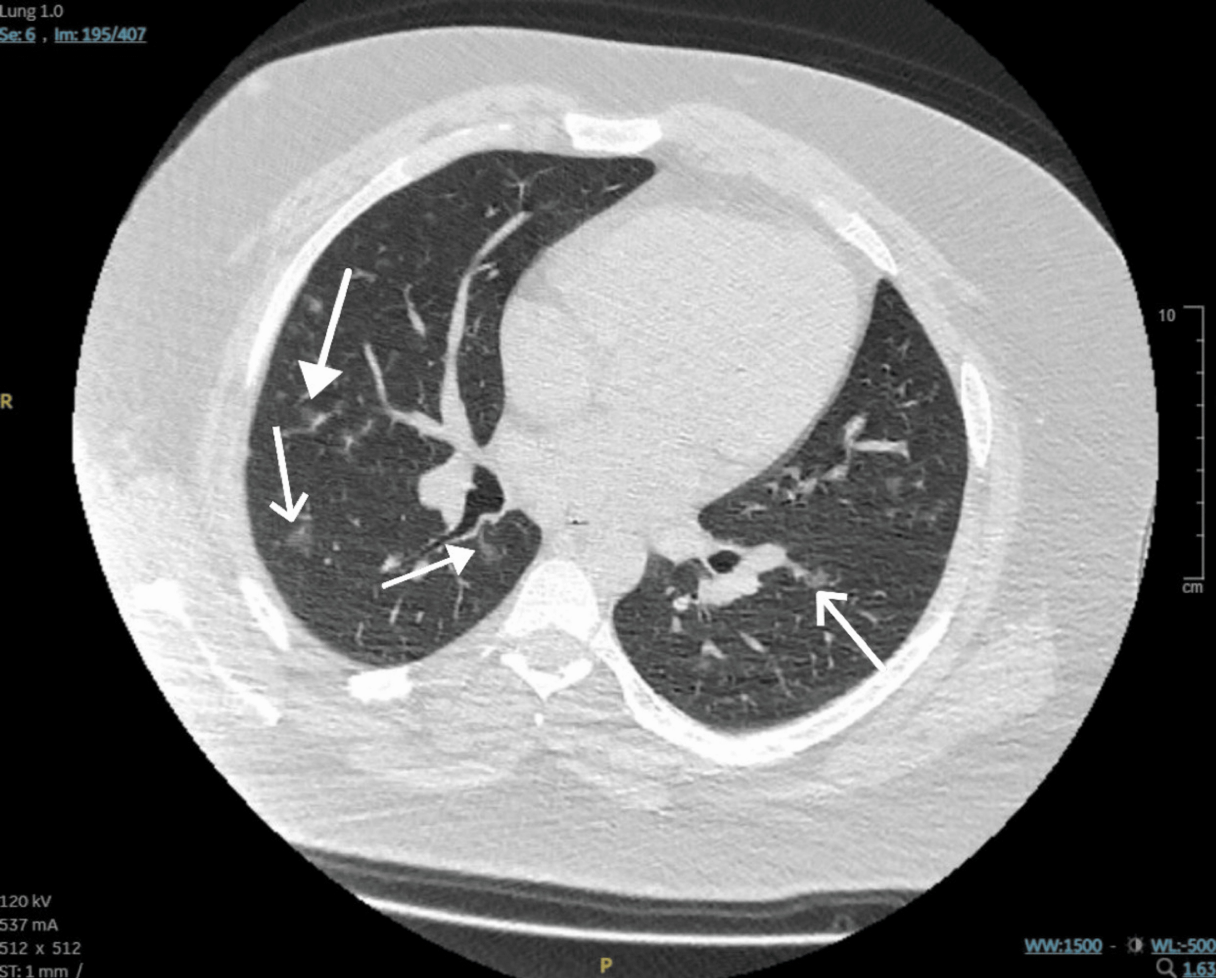
A CT scan of the chest displays areas of ground-glass opacity (white arrows), which are regions of hazy increased attenuation that do not obscure the underlying bronchial structures or pulmonary vessels.

Following improvement in inflammatory markers, the patient was switched to oral antibiotics. The same day, we got a call from the laboratory that the patient had tested positive for the anti-proteinase 3 antibody (63 IU/ml, reference range: 0-2 IU/ml). Positive anti-proteinase 3 antibody suggested a diagnosis of GPA. Therefore, a CT scan of the chest with contrast was requested, which revealed bilateral ground glass opacities suggestive of GPA (Figure 4). Additionally, the patient’s urine albumin creatinine and urine protein creatinine ratios were raised on further investigations. The urine albumin creatinine ratio was 167.6 g/mol (reference range: 0-3 g/mol), while the urine protein creatinine ratio was 310 mg/mol (reference range: 0-50 mg/mmol).

A formal diagnosis of GPA was made based on the clinical features and results of the tests. Respiratory and rheumatology departments' opinions were sought. The patient was started on a tapering course of high-dose prednisolone 90 mg once daily along with a proton pump inhibitor cover. The rheumatology department planned rituximab infusions as per the European Alliance of Associations for Rheumatology (EULAR) 2022 guidelines for organ-threatening disease given the patient’s renal and respiratory involvement.

The patient was assessed in the rheumatology clinic and counseled regarding rituximab treatment in the presence of his mother. He had initial difficulty understanding the risks and benefits of immunosuppression; however, after several discussions with the rheumatology consultant and the specialist nurse, he finally felt confident enough to consent to treatment. A psychology input would have been helpful, but unfortunately, we do not have access to this service in our Trust.

The patient has completed one course of rituximab with methylprednisolone, and the plan is for six monthly infusions over the next two years before switching to mycophenolate mofetil or azathioprine for maintenance therapy.

## Discussion

Granulomatosis with polyangiitis, previously known as Wegener granulomatosis, is an ANCA-associated vasculitis. This rare, multi-systemic disorder primarily affects small blood vessels and is characterized by granulomatous inflammation, pauci-immune necrotizing glomerulonephritis, and vasculitis, which result in endothelial injury and tissue damage [5]. The disease has an estimated incidence of 0.4 to 11.9 cases per million person-years [5]. Granulomatosis with polyangiitis occurs equally in males and females, typically presenting between the ages of 45 and 65, with a higher prevalence among the Caucasian population. The incidence appears to have been increasing in recent decades, likely due to heightened awareness and the development of ANCA testing [5].

The exact mechanisms of the disease are not well understood, but it is believed to be caused by multiple factors. Various immune system abnormalities lead to the excessive production of autoimmune antibodies, specifically cytoplasmic ANCA (c-ANCA) [6]. These antibodies target a protein called proteinase 3 (PR3), triggering neutrophil-mediated vascular damage. In GPA, the primary manifestation is vasculitis, which may or may not include granuloma formation, as observed in skin lesion histology. The necrosis of blood vessel walls is driven by inflammatory cytokines and other mediators induced by ANCAs, as well as heightened T-cell activity, especially involving CD4+ T-cells, which is associated with granuloma formation [6].

Ninety percent of GPA patients experience upper respiratory tract involvement. Initial symptoms often include issues like nasal and sinus pain, sinus congestion, purulent nasal discharge, nasal ulcerations, nosebleeds, and otitis media. The disease can also affect the lower respiratory tract, causing symptoms like cough, hemoptysis, dyspnea, and occasionally pleuritic chest pain. At the time of diagnosis, renal involvement is observed in only 10%-20% of cases, but within two years, 80% of patients develop glomerulonephritis. The most frequent form is rapidly progressive crescentic glomerulonephritis, which can lead to chronic kidney disease or end-stage renal disease [7].

The incidence and prevalence of ASD are on the rise [8]. Those individuals frequently experience various comorbid medical conditions, although the specifics and prevalence of these conditions are not well defined [8]. Medical disorders have often been overlooked in people with ASD, partly because obtaining a thorough medical history and conducting a physical examination can be challenging in patients who are often nonverbal and may exhibit behaviors that complicate detailed assessments. Nevertheless, recognizing and identifying these disorders is becoming increasingly important [8].

Autism spectrum disorder shares numerous characteristics commonly seen in autoimmune disorders, including a strong familial tendency and associations with immune system abnormalities. The genetic predisposition to ASD is complex and likely polygenic, while environmental factors can increase or modify the risk, and there are notable gender differences. The epidemiological connections between autoimmunity and ASD are compelling and have prompted several researchers to explore links between biologically-based autoimmune conditions and behaviorally-defined ASD. The initial studies suggesting a possible autoimmune connection to ASD date back to a 1971 case report, which explained a case of a child with ASD who also had a significant family history of autoimmune disorders [4]. 

The case presented here exemplifies the diagnostic complexities posed by GPA, particularly in the context of comorbid neurodevelopmental disorders such as ASD. Diagnosing GPA in this patient was particularly challenging due to the presence of atypical systemic symptoms and the coexistence of ASD and depression. The presentation of acute joint pain, petechial rash, and respiratory symptoms initially misled the diagnostic process to infection rather than an autoimmune pathology, emphasizing the importance of maintaining a high index of suspicion for rare autoimmune conditions in patients with complex medical histories and with family history of autoimmune diseases.

Furthermore, the multidisciplinary approach adopted in this case emphasizes the necessity of a well-rounded management strategy tailored to the patient's unique needs. Effective follow-up management of GPA requires collaboration among various specialties, including rheumatology, respiratory medicine, and nephrology, to ensure comprehensive care and optimize treatment outcomes. In the presence of neurodevelopmental disorders, input from a clinical psychologist may enhance the patient’s concordance with treatment and engagement with clinic appointments.

Before regulatory approvals in 2011 for using rituximab with glucocorticoids for treating patients with GPA, the standard-of-care treatment (since the 1970s) for severe disease was cyclophosphamide with glucocorticoids [9]. Rituximab is an anti-CD20 monoclonal antibody that targets and depletes CD20+ B cells and is approved for treating GPA [9]. In combination with glucocorticoids, rituximab is recommended by the EULAR as an alternative to cyclophosphamide for inducing remission of new-onset, organ- or life-threatening GPA [9]. Rituximab is also effective for maintaining remission in patients with GPA [9]. Cyclophosphamide treatment has implications concerning fertility in young patients, and if this treatment is considered, it is important to have this conversation with patients [10].

However, despite advances in treatment options, the challenges posed by ASD in treatment adherence remain significant. The patient's autistic traits may hinder engagement with treatment regimens, necessitating innovative approaches to enhance compliance and improve outcomes.

## Conclusions

This case brings to light the diagnostic and therapeutic challenges faced when managing GPA in patients who also have neurodevelopmental and psychiatric disorders. Such comorbidities can complicate the clinical presentation, making it more difficult to accurately diagnose GPA at the early stages and determine the most effective treatment plan. The overlapping symptoms of neurodevelopmental and psychiatric disorders can mask or mimic the signs of GPA, leading to potential delays or misdiagnosis. Autoimmune conditions and depression have many somatic symptoms in common, which makes it more challenging to diagnose GPA.

Treatment strategies for GPA may need to be adjusted to account for the unique needs and vulnerabilities of patients with these comorbid conditions. Crucially, a multidisciplinary approach should be adopted in such cases that involve collaboration among various healthcare professionals. This collaborative effort ensures a comprehensive evaluation and management plan that addresses the patient's medical and mental health needs. Finally, this case highlights the necessity for increased awareness among healthcare providers about the complexities of diagnosing and managing GPA in patients with mental issues.
